# Cycloidal CT with CNN-based sinogram completion and in-scan generation of training data

**DOI:** 10.1038/s41598-022-04910-y

**Published:** 2022-01-18

**Authors:** Daniël M. Pelt, Oriol Roche i Morgó, Charlotte Maughan Jones, Alessandro Olivo, Charlotte K. Hagen

**Affiliations:** 1 Leiden Institute of Advanced Computer Science, Leiden University, Niels Bohrweg 1, 2333 CA Leiden, The Netherlands; 2 Department of Medical Physics and Biomedical Engineering, University College London, Malet Place, London, WC1E 6BT UK

**Keywords:** Computational science, Imaging techniques, X-rays

## Abstract

In x-ray computed tomography (CT), the achievable image resolution is typically limited by several pre-fixed characteristics of the x-ray source and detector. Structuring the x-ray beam using a mask with alternating opaque and transmitting septa can overcome this limit. However, the use of a mask imposes an undersampling problem: to obtain complete datasets, significant lateral sample stepping is needed in addition to the sample rotation, resulting in high x-ray doses and long acquisition times. Cycloidal CT, an alternative scanning scheme by which the sample is rotated and translated simultaneously, can provide high aperture-driven resolution without sample stepping, resulting in a lower radiation dose and faster scans. However, cycloidal sinograms are incomplete and must be restored before tomographic images can be computed. In this work, we demonstrate that high-quality images can be reconstructed by applying the recently proposed Mixed Scale Dense (MS-D) convolutional neural network (CNN) to this task. We also propose a novel training approach by which training data are acquired as part of each scan, thus removing the need for large sets of pre-existing reference data, the acquisition of which is often not practicable or possible. We present results for both simulated datasets and real-world data, showing that the combination of cycloidal CT and machine learning-based data recovery can lead to accurate high-resolution images at a limited dose.

## Introduction

X-ray computed tomography (CT) has been an essential diagnostic device in medicine for decades, but more recently it has also emerged as a key tool for the non-destructive characterization of samples in biomedical research and other disciplines^1^. The breadth of the application space of CT stems in part from the ability to access spatial resolutions ranging from the mm down to the nm scale^2^. However, resolution is typically a pre-fixed property of a CT scanner, which depends on the characteristics of the x-ray source (namely the focal spot size) and of the x-ray detector (namely its point spread function, PSF)^3^. For example, most micro-CT machines allow changing the magnification (specifically, the position of the sample between source and detector), but there is a specific sample position (i.e. magnification value) that maximises resolution. This means resolution cannot always be adjusted to the needs of the sample without switching to specialised, high-resolution scanners. Moreover, increasing the resolution typically leads to longer scan times and to a higher dose of radiation being delivered to the sample, at least if no loss in the signal-to-noise ratio (SNR) is to be suffered^3^, which is problematic when scanning dose-sensitive samples or performing in vivo studies. As such, a key challenge in computed tomography is to obtain accurate high-resolution images using low-dose acquisition protocols.

Recently, cycloidal CT^4^ was proposed as a path towards solving this challenge. In cycloidal CT, the sample is illuminated by an array of x-ray beamlets generated by a mask, and translated within the in-slice plane *simultaneously* with being rotated; if the translation is unidirectional, each point in the sample follows a cycloidal trajectory (hence the name). Provided that the beamlets are sufficiently narrow and well separated, spatial frequencies that would otherwise be lost due to blurring by an extended source focal spot and/or detector point spread function (PSF) can now contribute to the image^5^. On the other hand, the scanner layout creates an undersampling problem, as sample areas located in between beamlets are not inspected. As a result, sinograms are highly incomplete, making the recovery of the missing entries an important step in the image reconstruction pipeline. It has been shown that interpolating incomplete cycloidal sinograms with bicubic splines, followed by filtered back projection (FBP) tomographic reconstruction, can lead to images similar in resolution to those reconstructed from complete sinograms, at a lower radiation dose^4^.

As such, cycloidal CT may be considered part of a larger class of methods whereby CT data are undersampled in an effort to save dose. A common approach is to acquire fewer projections than strictly necessary^6^. Image reconstruction is then often based on algebraic methods, where regularisation is applied to compensate for the data incompleteness^7^. Alternatively, the projections themselves can be undersampled by blocking out parts of the x-ray beam before it hits the sample; such intra-projection undersampling has been applied in the context of compressed sensing^8–11^. A further common approach for reducing dose is to decrease the exposure per sampled data point. Cycloidal CT also makes use of intra-projection undersampling, but it does not rely on compressed sensing-type data processing. It does though share similarities with other methods^9,12,13^ that employ a structured x-ray beam and non-standard sampling schemes, but with the unique difference that resolution can be increased and, as explained in the “Cycloidal CT” section, x-ray phase contrast accessed at the same time^14^.

In this paper, we report on applying machine learning to the sinogram completion task in cycloidal CT, with the aim of reconstructing high quality images from incomplete (low-dose) data, and demonstrating that bicubic interpolation (which was applied previously) can be outperformed. Over recent years, machine learning has seen a surge in application in the context of (standard) CT^15–27^. Convolutional neural networks (CNNs)^28–34^ have been applied as part of the tomographic image reconstruction process^18^, thereby improving the reconstruction quality, or as a post-processing tool to improve the quality of CT images after they have been reconstructed^15–17^. In addition, several papers propose using CNNs to recover missing parts of sinograms before reconstruction in low-dose or limited-angle settings^19–24^. However, most CNNs applicable to CT, especially those aimed at recovering missing information, must be trained on large sets of high-quality reference images (e.g., in the thousands) of similar samples to the one under investigation, acquired under similar conditions. In many cases, the acquisition of such training data can be either impossible or impractical, because it may be time-consuming or labour intensive, both in experimental and processing time, or the sample may be unique or dose-sensitive. In recent years, self-supervised learning, an approach that does not require any additional reference data, has shown promising results in tomographic reconstruction^26,27^. However, these methods are typically only applicable to the denoising of reconstructed images and rely on specific mathematical assumptions about the acquired data which makes them not directly usable for the sinogram completion task in cycloidal CT.

As an alternative approach, we here propose to acquire training data *as part of each scan*, by interleaving the acquisition of a few complete (“high dose”) projections with the incomplete (“low-dose”) cycloidal ones. This is made possible by the flexible nature of cycloidal data acquisition, as explained in the “Methods” section. Such an in-scan acquisition of training data has the advantage of not relying on large sets of pre-existing reference images, thus being suitable to any sample and, consequently, widely applicable. For efficient practical use of our proposed approach, it is important that only a few high-dose projections are required for accurate training, resulting in small training sets. Therefore, we propose to employ a Mixed-Scale Dense (MS-D) CNN^31^, which typically requires fewer intermediate images and learned parameters than other popular CNNs^28–30^ to achieve accurate results, making it well-suited for accurately learning from relatively small training sets.

This paper is organised as follows. In the “Background” section, we briefly describe the two main components of our approach: cycloidal CT and convolutional neural networks. In the “Methods” section, we introduce our proposed approach to data acquisition and training CNNs for cycloidal CT. In the “Simulations” section and “Experimental results” section, we demonstrate our approach’s performance on samples with different levels of complexity. Using simulated data, the network output is compared to images obtained through other common dose reduction strategies, including their processing with other popular CNNs. Experimentally, we show that the MS-D network can restore cycloidal sinograms acquired in both attenuation and phase contrast modes. Finally, we summarize our approach and give some concluding remarks in the “Discussion and conclusion” section. Our paper builds on a brief preliminary publication^35^ by (a) expanding the range of test samples and experimental conditions (now including phase contrast), (b) providing a wider comparison against existing methods, (c) investigating the amount of training data required and (d) demonstrating our method’s performance on flyscan data.

## Background

### Cycloidal CT

**Figure 1:**
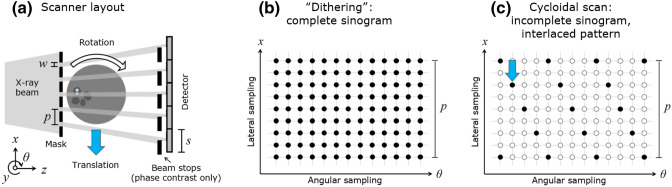
(**a**) Scanner layout (not to scale and extending uniformly into the *y*-direction), where *p* is the mask period and *w* is the aperture width of the mask; (**b**) Sinogram sampling pattern for a dithered acquisition, and (**c**) for a cycloidal acquisition. The sinogram grids are shown for a subset of angles and one mask period. The filled circles represent the sampled data points, while the empty circles represent the ones not sampled. This figure is adapted from ^35^.

The experimental layout of a cycloidal CT scanner^4^ is shown in Fig. 1a. The x-ray beamlets, created by apertures of width *w*, are typically a few $\mu$m to tens of $\mu$m wide and extend uniformly in the *y*-direction. The mask period (*p*) matches the effective detector pixel size (*s*) or may be an integer thereof. The sample is placed immediately downstream of the mask. Provided that (a) *w* is smaller than the combined PSF of the projected source and the detector, scaled to the mask plane, and (b) the beamlets are well-separated, spatial frequencies that would otherwise be lost to the source/detector blur can now contribute to the image. The presence of the mask has another advantage: sensitivity to phase contrast can be achieved by adding an array of beam stops in front of the detector, in such a way that their edges partly intercept each beamlet. This transforms the scanner into an ‘edge illumination’ x-ray phase contrast imaging system^14,36^, where refraction of the beamlets changes the fraction of x-rays detected by each pixel. X-ray phase contrast imaging is known to provide an improved contrast-to-noise ratio (CNR) for samples that exhibit weak intrinsic x-ray attenuation, such as soft biological tissue, light plastics, or other low atomic number materials^37^.

On the downside, the use of a mask creates an undersampling problem. Typically, the ratio between *w* and *p* is in the region of 1:3 to 1:10, meaning that projections are sampled substantially below the Nyquist rate. ‘Dithering’, a procedure by which the sample is stepped along the lateral scanner direction (i.e. along *x* in Fig. 1a) in steps of $\le$ *w* for each projection and the acquired frames combined, can provide fully sampled sinograms (Fig. 1b). The disadvantage of dithering is that it requires high acquisition doses and long scan times. A solution to both disadvantages is provided by cycloidal CT^4^, translating the sample along the scanner’s *x*-axis *as it rotates*. In addition, cycloidal CT is compatible with flyscans, as the sample can be “roto-translated” continuously without interruption. Cycloidal sampling results in incomplete sinograms that must be restored before CT reconstruction, as only a single frame is acquired per projection rather than multiple ones as during dithering. However, the acquired data points correspond to an interlaced sinogram sampling pattern (as shown in Fig. 1c), which eases the recovery of high-resolution features.

### Convolutional neural networks

In previous implementations of cycloidal CT, the data recovery step, i.e. filling the missing entries in cycloidally sampled sinograms, was performed using bicubic splines interpolation^4^. Here, we propose to use a CNN for this task instead. A CNN can be described as a function $y=f_\phi (x)$, which takes an input image *x*, produces an output image *y*, and is characterized by a set of parameters $\phi$. In many popular CNN architectures, the number of parameters can be as large as several million. The purpose of training CNNs is to find values for the parameters such that the CNN performs the task that is required. A popular way of training CNNs is supervised learning, in which we assume that we have access to a training set of *n* representative input images $X=\{x_1, x_2, \dots , x_n\}$ and corresponding target output images $Y=\{y_1, y_2, \dots , y_n\}$. Correct values for the CNN parameters can then be found by iteratively minimizing the difference between the output images of the CNN and the target output images: $\phi ^{\star } = \text {argmin}_{\phi } \sum _{i=1}^n L(f_{\phi }(x_i), y_i)$, where *L* is a chosen loss function that measures the error between two images, e.g. the mean squared error. In practice, there are several important considerations when minimizing the loss function in supervised learning, for example introducing measures to avoid overfitting the CNN to the specific training image set. For brevity, we will not discuss such aspects in this paper, but rather refer to existing literature on these topics^38–40^.

## Methods

### Cycloidal data recovery using convolutional neural networks

**Figure 2:**
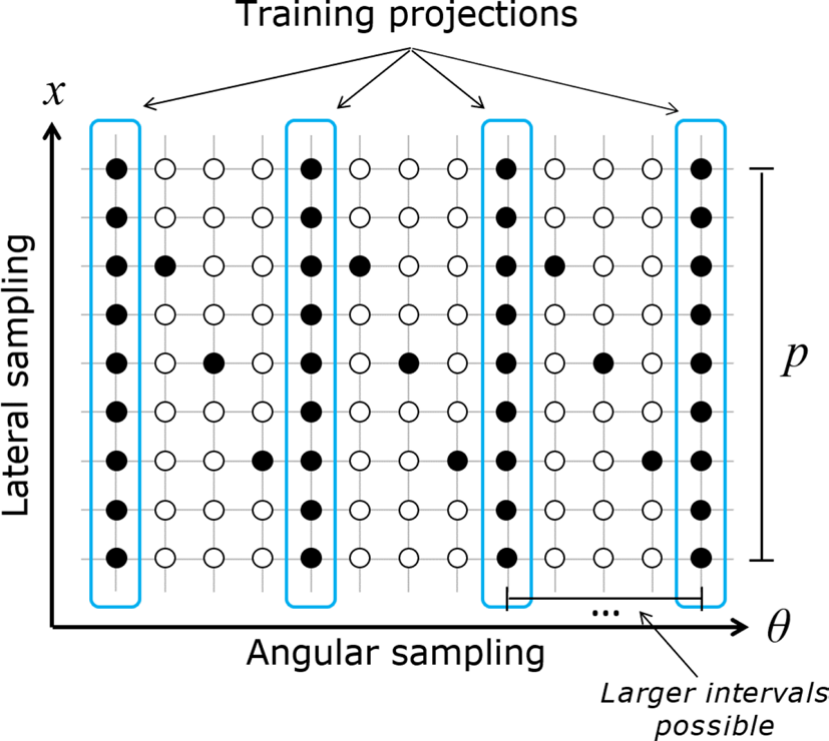
Cycloidal sinogram sampling pattern with training projections interleaved. The grid is shown for a subset of angles and one mask period (*p*). The filled circles represent the sampled data points, while the empty circles represent the ones not sampled. Note that the angular interval between training projections is typically substantially larger than indicated here for illustrative purposes. This figure is adapted from^35^.

**Figure 3:**
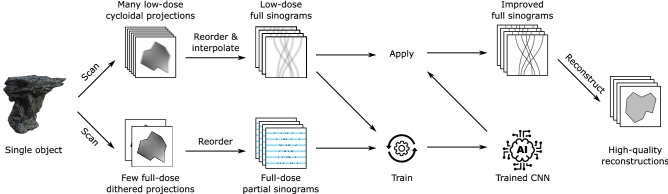
Schematic visualisation of the proposed training procedure.

Here, we introduce the main contribution of this paper: an approach to training CNNs for cycloidal CT that does not require the acquisition of high-dose reference projection data for a set of representative samples. Instead, in our approach, the training data are generated as part of each scan. This can be realised by interleaving the acquisition of a few complete projections with the incomplete cycloidal ones (i.e. by applying dithering at a subset of angles, which would correspond to a sampling pattern like the one shown in Fig. 2), or by acquiring a few dithered projections before or after acquiring the cycloidal projections. The network can then be trained in the following manner (see also Fig. 3). Let *n* be the number of axial slices of the sample for which sinogram data are available. First, the cycloidal sinograms are interpolated using bicubic splines. Next, the dithered projections are arranged into ‘partial’ sinograms, meaning that, while they are the same size as the interpolated cycloidal sinograms, they only contain data at those angles at which dithering was applied. For training purposes, we consider the *n* interpolated cycloidal sinograms (‘low-dose full sinograms’ in Fig. 3) the set of input images $X=\{x_1,x_2,\dots ,x_n\}$, while the *n* ‘partial’ sinograms (‘full-dose partial sinograms’ in Fig. 3) are considered the set of output images $Y=\{y_1, y_2,\dots ,y_n\}$ (the training target). Our training algorithm then minimizes the following loss function:

1 $$\begin{aligned} L_{cyc} \left( f_{\phi }(x_i), y_i \right) = \sum _{j\in D} \left( f_{\phi }(x_i)^j - y_i^j \right) ^2 \end{aligned}$$

Here, $x^j$ denotes the *j*-th pixel of image *x*, and the domain *D*, which defines the data points included in the sum, is restricted to those pixels for which the ‘partial’ sinograms include a measurement. Although we use an $\ell _2$ loss function in Eq. (1), other types of loss function could be used, potentially improving results^41^. However, since the loss function is computed on sinograms instead of reconstructed images in our approach, common reconstruction-based regularization terms such as Total Variation minimization might not be directly beneficial.

Once the network is trained, it can be applied to the interpolated cycloidal sinograms. This produces a set of restored sinograms (‘improved full sinograms’ in Fig. 3), which can be reconstructed into tomographic images. We note that this approach of training with ‘partial’ sinograms is enabled by the flexible nature of cycloidal data acquisition, making it possible to acquire both high-dose and low-dose parts of a single sinogram. While there is flexibility in choosing the angles at which to acquire the dithered projections, we have found that distributing them evenly across the total angular range yields the best results. The described training procedure leads to a unique CNN that is specific to the sample being scanned and the parameters of the particular scan. Note that the integration of dithered projections adds to the dose delivery to the sample and prolongs scans; however, in practice this can be very small, e.g. the additional training data can amount to as little as 1% of the complete sinogram (see the “Simulations” section).

### Choice of network architecture

While, in principle, any CNN capable of learning image-to-image mappings, such as a U-Net^28^, SegNet^29^ or DeepLab^30^ network, may be used to recover incomplete datasets, the sinogram recovery task in cycloidal CT has some specific challenges that can make it difficult to train and apply popular off-the-shelf CNNs, at least without dedicating additional effort to make them perform efficiently and accurately in practice. First, existing CNNs typically require large amounts of training data to produce accurate results (e.g. thousands of example images), while the amount of training data in our approach is limited to a few percent of all pixels of the sinogram images. Second, existing CNNs typically are not able to directly process large images due to their significant computer memory requirements (specifically, GPU memory), while cycloidal sinograms are usually relatively large (e.g. they contain more than a thousand rows and columns). A common solution to reduce memory requirements is to train and apply CNNs on smaller patches extracted from large images^19,22^, but for sinograms this approach could lead to suboptimal results because the small patches might lack important non-local contextual information and stitching network outputs together might introduce artifacts^23^.

As an alternative, in this work we propose to use the recent Mixed-Scale Dense (MS-D) CNN architecture^31^. In the following, we will give a brief description of the architecture, focusing on the advantages it provides for the specific task of data recovery in cycloidal CT. For more general details about the structure of MS-D networks, including mathematical definitions and comparisons with popular existing CNN architectures, we refer to^31^. MS-D networks differ from most existing CNNs in two key points: first, dilated convolutions are used exclusively to capture image features across multiple scales, instead of the commonly used scaling operations. Second, all intermediate images within the network are connected to each other, instead of only connecting images of successive layers. Both changes result in a CNN that requires fewer intermediate images and fewer learned parameters compared with other CNNs, and that can automatically adapt to different problems. In practice, this means that large images can be efficiently processed without running out of computer memory, and accurate training is possible with a limited amount of training data. These advantages have already proven effective for various non-cycloidal applications of CT in earlier work^17,25–27^, and make the MS-D CNN especially applicable to the task of data recovery in cycloidal CT. Recently, other CNN architectures were proposed that also make use of dilated convolutions and dense connections to capture multi-scale information^32–34^. However, such networks are typically not directly designed for solving the problems of accurate training with limited amounts of training data and handling large images efficiently, making it difficult to train and apply them in cycloidal CT without significant additional effort. In the “Simulations” section, we directly compare results between MS-D networks and other existing architectures.

## Simulations

Our approach was first tested on simulated CT data of a numerical “foam” sample, generated by removing 150000 randomly-placed non-overlapping spheres with varying sizes from a cylinder of a single material. Similar foam phantoms were used to compare reconstruction algorithms in earlier work^17^, showing that these phantoms are difficult to reconstruct accurately even with advanced regularized iterative methods such as TV minimization because of the combination of large-scale and fine-scale features. 1024 projections of the sample were simulated over 180 degrees using the foam_ct_phantom software package^42^, assuming a detector of 1024 by 1024 pixels and a parallel-beam acquisition geometry. 1024 sinograms were created from the projections, each one containing 1024 pixels by 1024 pixels. A cycloidal acquisition was simulated using an aperture width of one pixel, a mask period of eight pixels, and a sample movement of 3 pixels between subsequent projections. The aperture of eight was chosen as this matches that of the experimental apparatus described in “Experimental results” section, where the ratio between mask apertures and opaque areas is 1:8. Poisson noise was applied to the generated projections, using a virtual exposure time such that 1000 simulated photons passed through the sample for each detector pixel, with the sample absorbing roughly half of the photons on average.

Training projections (33, distributed evenly across the total angular range) were also extracted from the complete dataset. A 100-layer MS-D network with dilations between one and ten was trained using the approach described in “Cycloidal data recovery using convolutional neural networks” section. For training and applying networks we used the Python implementation accompanying^31^, available as an open-source package (https://github.com/dmpelt/msdnet). Out of the available sinograms, 922 (90%) were used for training the network, while the remaining 102 (10%) were used as a validation set to monitor performance. The network was trained for 48 hours using the ADAM algorithm^43^ with the default learning rate of 0.001, which corresponded to roughly 68 epochs. The network parameters were initialized using the approach described in^31^, and the parameters that resulted in the lowest mean squared error on the validation sinograms were stored for further processing. Note that we chose a training time of 48 hours to ensure convergence of the training process in all cases, while preventing overfitting by using a separate validation set to monitor performance, enabling a fair comparison between different networks. In practice, we observe that accurate results are often achieved after significantly shorter training times (i.e., after a few epochs), as evidenced by the results of Fig. 7 described below.

The flexible nature of the simulation provided the opportunity to carry out further tests for which experimental data were not readily available. Besides comparing cycloidal sinograms restored via the MS-D network to complete sinograms, we also evaluated our approach’s effectiveness against other established dose reduction methods: angular subsampling by omitting projections, and reducing the exposure time per projection. To simulate the former, all but every 8$^{\text {th}}$ projection were discarded from the complete dataset. For the latter, the number of simulated photons passing through the sample was reduced by a factor of eight from 1000 to 125 photons per detector pixel. We also compare results with a state-of-the-art machine learning approach specifically designed for processing low exposure time data^44^. To apply this approach, we trained a similar MS-D network as above to denoise the low exposure projections. 33 projections out of all available 1024 projections, equally distributed over the angular range, were used for training, in which the network input consists of a projection with 125 simulated photons and the training target consists of the corresponding projection with 1000 simulated photons. After training for 48 hours, the trained network was applied to all 1024 low exposure time projections to produce denoised projections, which can be reconstructed using standard tomographic algorithms. While these tests provide a broader picture, it is important to note that with a setup like the one shown in Fig. 1a fully sampled projections can only be acquired with dithering, even in angular subsampling and low exposure time settings, which significantly increases scan times and is incompatible with flyscans. In addition, the exposure time per projection can often only be reduced up to a certain point in experimental setups because of practical constraints such as fixed detector readout times and maximum sample rotation speeds.

**Figure 4:**
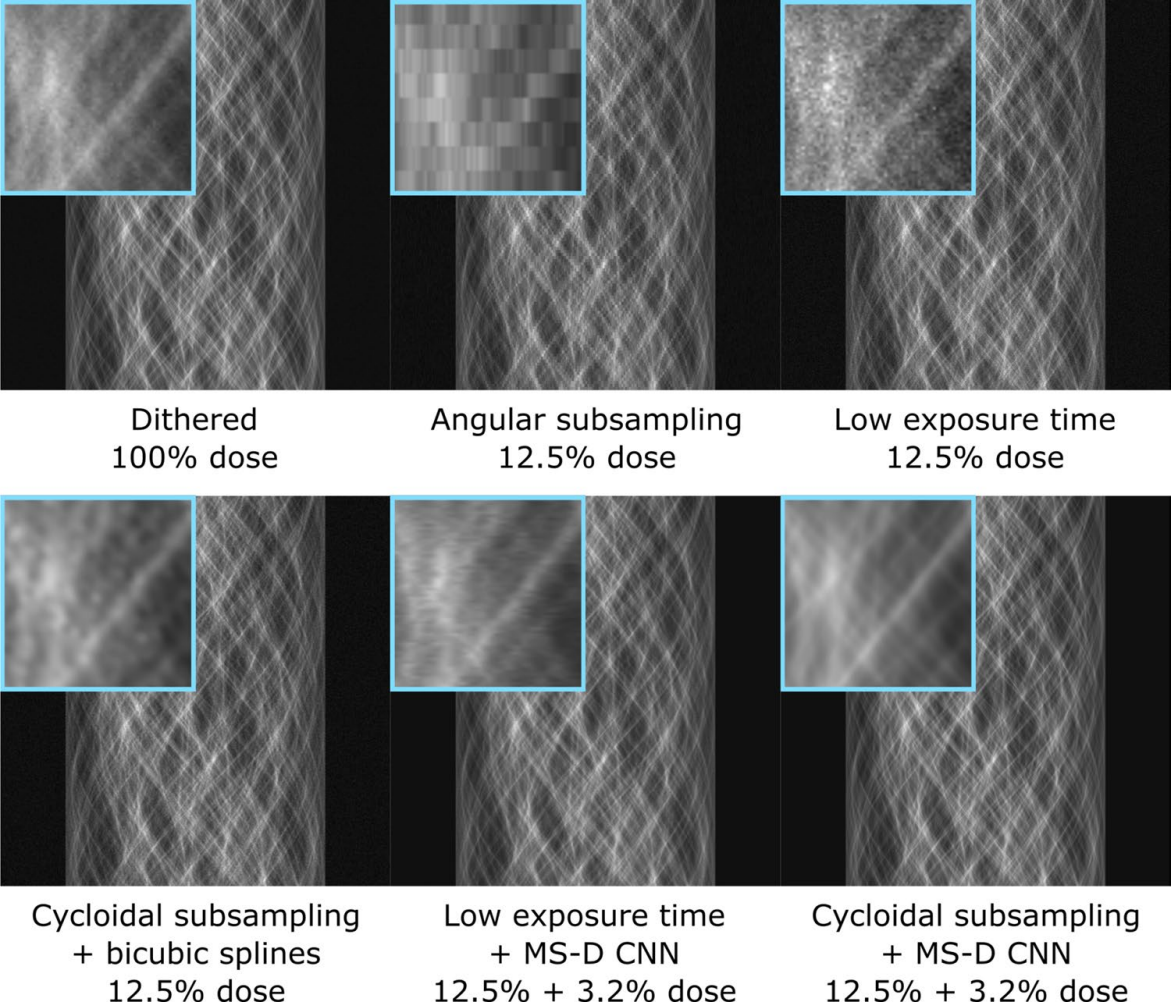
Simulated sinograms for the numerical foam sample, from left to right and top to bottom: complete sinogram, angular subsampling, low exposure time, cycloidal subsampling and bicubic splines interpolation, low exposure time data with projection denoising using a MS-D CNN^44^, cycloidal subsampling and MS-D CNN data recovery.

**Figure 5:**
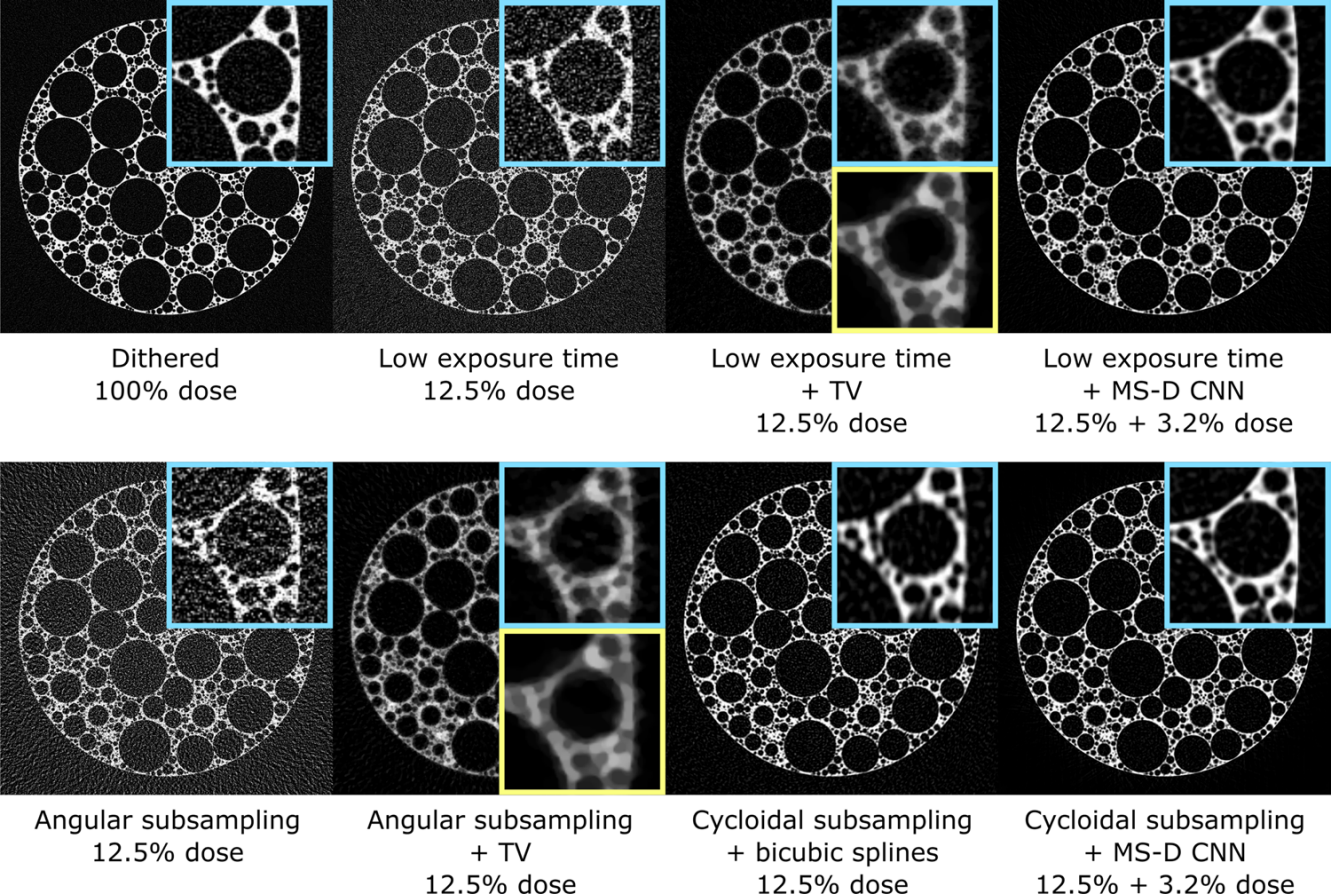
Tomographic images reconstructed from the simulated sinograms for the numerical foam sample, from left to right and top to bottom: reconstruction from complete data, from low exposure time data, from low exposure time data using TV reconstruction, from low exposure time data with projection denoising using a MS-D CNN^44^, from angularly subsampled data, from angularly subsampled data using TV reconstruction, from cycloidally subsampled data processed with bicubic splines interpolation, from cycloidally subsampled data processed with MS-D CNN data recovery. For each image, a small section is shown enlarged in the top right. For TV reconstruction, the parameter controlling the strength of the TV term was chosen such that the PSNR is maximized. In the bottom right of the TV reconstructions, the same small section is shown for a parameter choice that maximizes the MS-SSIM, showing the significant effect that this parameter has on the reconstructed image.

The resulting sinograms are shown in Fig. 4. In addition to the described scenarios, we have also included a cycloidal sinogram interpolated with bicubic splines but without applying the MS-D CNN. A complete dithered sinogram is shown for reference. The results show that angular subsampling removes fine details from the sinogram, while low exposure times result in noisy sinograms. Sinograms produced by cycloidal subsampling paired with bicubic splines interpolation retain more details than angular subsampling and low exposure times, but are somewhat blurry. Denoising low exposure time projections using a CNN produces sinograms with slight inconsistencies between rows, possibly because this approach does not ensure sinogram consistency during training. In contrast, our proposed combination of cycloidal subsampling paired with CNN recovery produces sharp and consistent sinograms that retain fine details. Tomographic images, reconstructed with FBP using the ASTRA toolbox^45^ and Total Variation minimization (TV) using the Chambolle-Pock algorithm^7^ implemented with the tomosipo package^46^, are shown in Fig. 5. The observations on performance are largely in line with those for the sinograms; generally the application of an MS-D CNN appears to provide the clearest images and suppresses background artefacts. Table 1 provides a quantitative comparison between the respective scenarios, based on calculating the peak signal-to-noise ratio (PSNR), Dice similarity coefficient^47^ (Dice) and the multiscale similarity index^48^ (MS-SSIM). The Dice scores are computed for reconstructions that are thresholded to produce images that are segmented into foreground and background pixels. In all cases, the metrics are computed in comparison with a ground truth image that is generated using the mathematical phantom shape definition.

**Table 1:** Quantitative comparison between different subsampling strategies based on the metrics PSNR, Dice and MS-SSIM.

	Dose (%)	PSNR	Dice	MS-SSIM
Complete dataset	100.0	18.38	0.957	0.743
Low exp.	12.5	9.59	0.725	0.497
Low exp. + TV	12.5	16.07	0.868	0.928
Low exp. + MS-D	15.7	18.55	0.920	0.871
Ang. subs.	12.5	8.11	0.642	0.410
Ang. subs. + bicubic	12.5	13.36	0.807	0.704
Ang. subs. + TV	12.5	14.99	0.798	0.910
Cyc. subs. + bicubic	12.5	17.32	0.903	0.710
Cyc. subs. + MS-D	15.7	19.19	0.925	0.928

Results are given for angular subsampling (Ang. subs.), low exposure time (Low exp.), and cycloidal subsampling (Cyc. subs.), with and without bicubic splines interpolation (bicubic), TV reconstruction (TV), or application of the MS-D CNN (MS-D). For TV reconstructions, the parameter controlling the strength of the TV term was chosen separately for each column, such that the metric of that column is maximized for the central slice.

**Figure 6:**
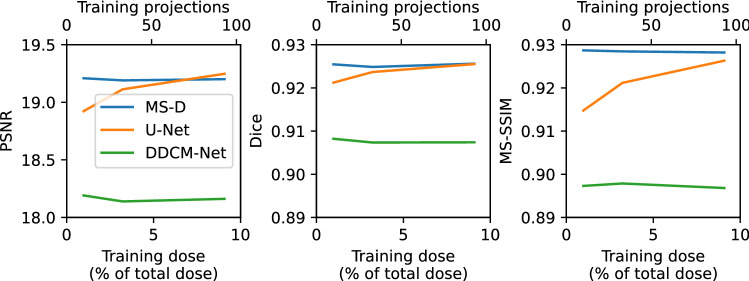
Quantitative comparison of images obtained after training MS-D CNNs, U-Net CNNs, and DDCM-Net CNNs using different numbers of training projections.

**Figure 7:**
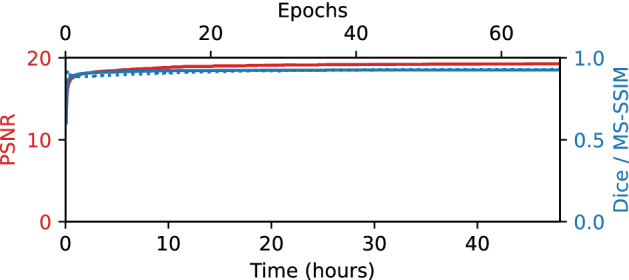
Accuracy metrics (PSNR, Dice, and MS-SSIM) for reconstructions of the central slice of the foam phantom, using cycloidal subsampling with an MS-D network at various points during the 48 hour training time.

Since the application of the MS-D network relies on the availability of training data, the overall amount of dose needed for image reconstruction is somewhat larger compared to the cases reconstructed without applying the network. To generate the specific results shown in Figs. 4 and 5 (MS-D CNN panels), the training data constituted 3.2% of the complete dataset. More generally, this gives rise to the question as to how many projections are required to train the network to a satisfactory level. We have carried out a preliminary investigation by processing the cycloidal sinograms again after training the network on a smaller (1%) and larger (9.7%) fraction of the complete dataset. We also performed the same experiments using the popular U-Net CNN architecture^28^ and the DDCM-Net CNN architecture^32^, which includes both dense connections and dilated convolutions similar to the MS-D architecture. Here, we used widely-used PyTorch^49^ implementations of U-Net (https://github.com/milesial/Pytorch-UNet) and DDCM-Net (https://github.com/samleoqh/DDCM-Semantic-Segmentation-PyTorch), and trained networks in a way identical to the MS-D networks described above. The images (not shown) were again analysed based on the PSNR, Dice and MS-SSIM. The results, shown in Fig. 6, reveal there is negligible difference between the three training dose settings for the MS-D network, indicating that the MS-D network is able to accurately learn from a limited number of training examples. In contrast, although U-Net trained with a large amount of data (9.7%) produces images with metrics similar to the MS-D networks, the accuracy of U-Net trained with fewer images is significantly lower. The metrics of images produced by DDCM-Net are significantly lower than those of the images produced by MS-D and U-Net, even with a large amount of training data. As a further test, we trained a DDCM-Net network with an even larger amount of training data (33.3%), which resulted in an improvement in metrics compared with training with fewer training images (PSNR: 18.31, Dice: 0.910, MS-SSIM: 0.907). These results indicate that U-Net and DDCM-Net CNNs can indeed have problems with overfitting in this setting, and that MS-D networks are a good fit for cycloidal data recovery. In Fig. 7, the accuracy metrics for reconstructions of the central slice using cycloidal subsampling and an MS-D network are shown as a function of the training time. These results show that most improvements by the CNN are achieved in the first few hours of training.

## Experimental results

### Experimental apparatus

Experimental data were acquired with two custom-designed imaging setups. The first one (‘system 1’) comprised a MicroMax-007 HF x-ray tube (Rigaku, Japan) with a rotating molybdenum anode, operated at 40 kV and 25 mA, resulting in a horizontal focal spot of approximately 70 $\mu$m (full width half maximum). The x-ray spectrum is polychromatic, with a mean energy of approximately 18 keV. The detector was the Pixirad-2 photon counter with a pixel size of 62 $\mu$m. The mask (Creatv Microtec, USA) had a 79 $\mu$m period and 10 $\mu$m apertures. The mask and detector were positioned at 1.6 m and 2.53 m from the source, respectively. With these distances, the mask period covered two detector pixel columns when projected to the detector, which is equivalent to using a detector with twice as large pixels horizontally. The second setup (‘system 2’) was operated in phase contrast mode. The system comprised the same x-ray tube as above, operated at 40 kV and 20 mA. The detector was a CMOS-based flat panel C9732DK-11 with 50 $\mu$m pixels from Hamamatsu (Japan). The mask was the same as the one used in ‘system 1’, but here placed approximately at 0.7 m from the source. The distance between source-to-detector was 0.875 m. To generate sensitivity to phase effects, a second mask was placed immediately in front of the detector; this ‘detector mask’ had an aperture width of 17 $\mu$m and a period of 98 $\mu$m. The detector mask functions as an array of partial beam stops, which, as explained in the “Cycloidal CT” section, allows sensing the beamlets’ refraction. The two masks were aligned with a relative lateral offset of 9 $\mu$m.

### Tests on attenuation contrast images

Our approach was first tested on attenuation data acquired with ‘system 1’. The sample was a piece of chicken bone, fixed in formalin and placed in a cylindrical plastic container of approximately 8 mm diameter. To prevent movement of the sample, it was surrounded by agarose. A complete dataset was acquired by means of dithering, which involved step-scanning the sample in eight steps (10 $\mu$m each) at each rotation angle; since $w \approx p/8$, this was the number of steps required to ensure that in each projection the sample was fully illuminated. The sample was rotated in 0.2 degree angular steps over 180 degrees, corresponding to the acquisition of 900 projections (900 $\times$ 8 = 7200 frames). The exposure time per frame was 2 s. Cycloidal sinograms were generated by subsampling the complete dataset in the same manner as for the simulated data, i.e. by discarding all but every 8$^{\text {th}}$ pixel column from each projection, with an offset of three pixel columns between angles (the offset corresponds to three dithering steps, i.e. a 30 $\mu$m lateral displacement of the sample). For step-and-shoot acquisitions, this approach is equivalent to performing a cycloidal scan where only a single frame is acquired per projection, but the sample is translated by 30 $\mu$m between angles. Training projections (29, distributed evenly across the total angular range) were also extracted from the dithered dataset. In the same manner as for the simulated data, a 100-layer MS-D network was trained with bicubic splines interpolated cycloidal sinograms as the network input, and ‘partial’ sinograms containing only those dithered projections as the training target. Out of the available sinograms, 270 (90%) were used for training the network, while the remaining 30 (10%) were used as a validation set to monitor performance. The trained network was applied to the bicubic splines interpolated cycloidal sinograms to produce improved interpolated sinograms which were reconstructed using the FBP method of the ASTRA toolbox.

**Figure 8:**
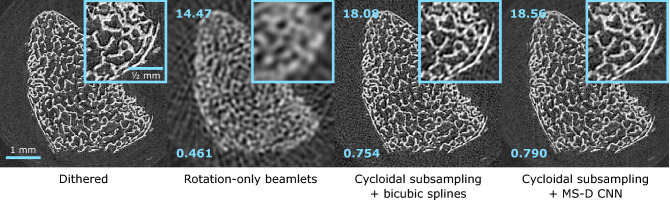
Tomographic images of a chicken bone (attenuation contrast; step-and-shoot acquisition). From left to right: image reconstructed from a complete (i.e. dithered) sinogram, an incomplete rotation-only beamlet sinogram interpolated with bicubic splines, an incomplete cycloidal sinogram interpolated with bicubic splines, an incomplete cycloidal sinogram processed with the MS-D CNN. The PSNR (top left) and MS-SSIM (bottom left) with respect to the dithered image are shown for each low-dose image.

Figure 8 shows tomographic images reconstructed from interpolated cycloidal sinograms processed with and without the MS-D network. The network produces a better image quality than interpolation alone, both in terms of definition and a reduction of background artefacts. The dithered image is shown for reference. For comparison, a reconstruction from a ‘rotation-only’ beamlet sinogram is shown as well. This sinogram is obtained by discarding all but every 8$^{\text {th}}$ pixel column from each projection, similar to the cycloidal sinogram, but without using an offset between angles. Such an acquisition approach corresponds to using the same beamlet system as cycloidal scans, but only rotating the sample during acquisition (i.e., without simultaneous translation), hence the name ‘rotation-only’^4^.

### Tests on phase contrast images

X-ray phase contrast scans were performed with ‘system 2’. The sample was a custom-built phantom made from polyethylene spheres with a 425-500 $\mu$m diameter, placed in a 3 mm plastic straw. A dithered dataset was acquired using the same scan parameters as above (8 dithering steps, 900 projections, 180 degree angular range, 2 s exposure per frame). The cycloidal subsampling was also performed in the same manner. The MS-D CNN was trained on 29 projections extracted from the dithered dataset. Out of the available sinograms, 137 were used for training the network, while the remaining 15 were used as a validation set to monitor performance. After the cycloidal sinograms were processed by the trained network, phase retrieval was applied in order to convert the refraction signal, which in the images manifests as edge enhancement, into area contrast. We used the ‘single-image’ phase retrieval method^50^ by which a tailored low-pass filter is applied to the sinograms. The method is derived by assuming a linear relationship between the real and imaginary parts of the sample’s complex refractive index, and is an adaptation of the widely used Paganin algorithm for free space propagation x-ray phase contrast imaging to the edge illumination technique^51^.

**Figure 9:**
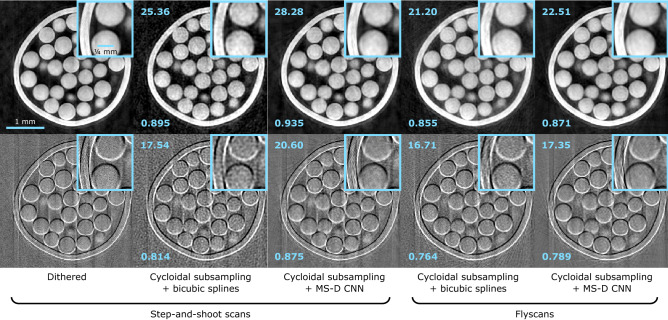
Tomographic images of 425–500 $\mu$m diameter polyethylene spheres (phase contrast acquisition). From left to right: images reconstructed from complete (i.e. dithered; step-and-shoot) sinograms, cycloidal sinograms interpolated with bicubic splines (step-and-shoot), cycloidal sinograms processed with the MS-D CNN (step-and-shoot), cycloidal sinograms interpolated with bicubic splines (flyscan) and cycloidal sinograms processed with the MS-D CNN (flyscan). The top row shows phase retrieved images, while the bottom row shows what these images would look like if phase retrieval were not applied. The PSNR (top left) and MS-SSIM (bottom left) with respect to the dithered images are shown for each low-dose image.

Tomographic images are shown in Fig. 9 (step-and-shoot panels). The top row shows phase retrieved images; for comparison, the bottom row shows how these images would look like if phase retrieval was not applied. In the non-retrieved images, as a consequence of their weak x-ray attenuation, the polyethylene spheres can be seen only via the refraction signal highlighting their edges. The cycloidal images processed with the MS-D CNN are visually indistinguishable from the dithered image.

### Demonstration of compatibility with continuous scanning (flyscans)

The scans described in the “Tests on attenuation contrast images” section and “Tests on phase contrast images” section were performed in step-and-shoot mode. In that case, a cycloidal dataset can be interpreted as a subset of a dithered dataset. However, cycloidal CT can also be implemented in continuous mode, i.e. as a flyscan, by translating and rotating the sample without interruption. Flyscans have the advantage that overheads caused by stop-starting the motors are eliminated. Depending on the detector, dead times needed for read-out can be negligible (e.g., the Pixirad-2 in ‘system 1’ has a negligible read-out time with frames rates in excess of 100 fps). If this is the case, scan time is determined by the exposure time alone. On the downside, the continuous sample motion may introduce a degree of blur, causing a small loss in resolution. Since a continuous cycloidal dataset cannot be generated by subsampling a dithered one, we have performed a standalone flyscan of the sphere phantom using ‘system 2’. The sample was rotated at a speed of 0.1 degrees/s and, simultaneously, translated laterally at a speed of 10 $\mu$m/s. 900 projections were acquired with an exposure time of 2 s each; hence the sample covered an angular interval of 0.2 degrees and a lateral distance of 20 $\mu$m between projections. In total, the sample was rotated over 180 degrees and translated over 1.8 cm. A few extra frames at the beginning and end of the acquisition were also collected to mitigate the effect of motor acceleration and deceleration, but they were discarded before further processing. The fact that the sample was in a different lateral position at the end of the scan compared to where it started out from meant that projections needed to be “shifted back” when being reassembled into sinograms to restore a meaningful sample geometry (a detailed description of this procedure can be found elsewhere^52^). As explained above, for flyscans the dithered training projections can be acquired immediately before or after the cycloidal scan. In our particular example, training projections were extracted from a previously acquired dithered dataset and registered to the flyscan projections using cross correlation^53^. Training was again performed according to “Cycloidal data recovery using convolutional neural networks” section and the trained network applied to the interpolated cycloidal sinograms. Tomographic images, reconstructed with FBP, are shown in Fig. 9 (flyscan panels). No notable degradation of image quality is observed relative to the step-and-shoot results, although the image quality metrics do indicate differences, potentially caused by slight misalignments between the flyscan and step-and-shoot acquisitions.

## Discussion and conclusion

In this work, we have explored the combination of cycloidal CT, a low-dose acquisition strategy for high-resolution micro-CT, with CNN-based data recovery. The task of the CNN was to recover missing entries in cycloidal datasets, based on learned relationships between interpolated incomplete sinograms and fully-sampled projections. We have selected the MS-D network architecture, as it was shown previously to reduce the computational burden and the number of training images required to process large scale CT data; both attributes are of relevance when processing cycloidal CT data. So far, MS-D networks had been used to improve the quality of tomographic images post reconstruction. Here, the network was adapted to, and applied in, the sinogram domain. Additional novelty lies in the training; for step-and-shoot scans, we have shown that the network can be successfully trained on a few dithered projections, evenly distributed across the angular range. For flyscans, the network can be trained on projections acquired before or after the scan. In this sense, a unique network is trained for each scanned sample and set of experimental parameters.

Application of the trained network to bicubic splines interpolated sinograms was found to provide sharper images with fewer background artefacts than interpolation alone. A comparison of several image quality metrics (PNSR, Dice, MS-SSIM) has supported this: all of them were found to be superior for the MS-D network. By means of simulated data, we have further investigated how cycloidal CT combined with the MS-D compares against other dose reduction methods, angular subsampling and exposure time reduction, again showing superior performance, even when using other approaches commonly used to handle incomplete data (e.g., iterative reconstruction with TV regularisation). Note that we did not include the case of angular subsampling and MS-D based data recovery into the comparison, as in that case there is no meaningful way of training the network according to the training approach proposed in this paper. This would require learning the relationship between interpolated angular subsampled sinograms and sinograms for which all angular views are available, the latter indeed being the entire dataset hence there would be no need to deal with angular subsampling in the first place.

The performance of the MS-D network applied to cycloidal CT data was also compared to that of other popular CNNs (U-Net and DDCM-Net). The purpose of this was (a) to compare the results, and (b) to understand the requirements in terms of training data. It should be noted that our approach to training increases the overall amount of data and, thus, scan time and dose; hence, the training dataset should be kept as small as possible. Our results suggest that the MS-D network can indeed cope well with small amounts of training data (notably, training on as little as 1% of the complete dataset can already lead to high-quality images), outperforming the other tested networks in this regard.

Before concluding, we would like to mention several limitations of the presented work. First, it should be noted that, while the numerical and experimental samples used to test our approach exhibit structural complexity, they are relatively homogeneous in terms of material composition. The performance of the method on multi-material and/or very low-contrast samples has not been investigated. Second, the exact validity limits of the method have not yet been explored, including how the method performs when using different scanning setups or with more severe degrees of sub-sampling. Third, the possibility of continuous scanning resulting in unwanted sample motion has not been studied. Fourth, the experimental results shown were obtained for rather small samples and relatively long source-to-detector distances, enabling treating the acquisition geometry as a parallel-beam system. Indeed, to obtain the results shown, we have applied FBP without any further consideration of the conical shape of the x-ray beam. We have not yet investigated in detail how the cycloidal CT method performs under a fan or cone beam tomographic geometry. We are planning to investigate all of the above as part of future work.

In conclusion, cycloidal CT combined with CNN-based sinogram completion enables in-scan generation of training data, and can produce high-quality images from a reduced number of acquired data points, demonstrating a superior performance compared with previously applied data processing methods. We anticipate that the improvements in image quality will make a notable difference when visualising samples that contain faint and/or minute features. This makes it a suitable candidate for applications that rely on a low radiation dose delivery, or short scan times, e.g., to accommodate a high sample throughput. The fact that large sets of pre-existing images are not required for training the network makes the proposed approach widely applicable.

## References

[1] Withers PJ (2021). X-ray computed tomography. Nat. Rev. Methods Primers.

[2] Burnett T, Withers P (2019). Completing the picture through correlative characterization. Nat. Mater..

[3] Buzug T (2008). Computed Tomography; From Photon Statistics to Modern Cone-Beam CT.

[4] Hagen, C. K., Vittoria, F. A., iMorgó, O. R., Endrizzi, M. & Olivo, A. Cycloidal computed tomography. Phys. Rev. Appl. **14**, 014069 (2020).

[5] Diemoz PC, Vittoria FA, Olivo A (2014). Spatial resolution of edge illumination x-ray phase-contrast imaging. Opt. Exp..

[6] Rangayyan R, Dhawan AP, Gordon R (1985). Algorithms for limited-view computed tomography: an annotated bibliography and a challenge. Appl. Opt..

[7] Sidky EY, Jørgensen JH, Pan X (2012). Convex optimization problem prototyping for image reconstruction in computed tomography with the chambolle-pock algorithm. Phys. Med. Biol..

[8] Choi, K. & Brady, D. J. Coded aperture computed tomography. In *Adaptive Coded Aperture Imaging, Non-Imaging, and Unconventional Imaging Sensor Systems*, vol. 7468, 74680B (International Society for Optics and Photonics, 2009).

[9] Koesters, T., Knoll, F., Sodickson, A., Sodickson, D. K. & Otazo, R. Sparsect: interrupted-beam acquisition and sparse reconstruction for radiation dose reduction. In *Medical Imaging 2017: Physics of Medical Imaging*, vol. 10132, 101320Q (International Society for Optics and Photonics, 2017).

[10] Cuadros AP, Arce GR (2017). Coded aperture optimization in compressive x-ray tomography: a gradient descent approach. Opt. Exp..

[11] Mojica E, Pertuz S, Arguello H (2017). High-resolution coded-aperture design for compressive x-ray tomography using low resolution detectors. Opt. Commun..

[12] Cho, S., Lee, T., Min, J. & Chung, H. Feasibility study on many-view under-sampling technique for low-dose computed tomography. *Opt. Eng.* **51**, 080501 (2012).

[13] Abbas S, Lee T, Shin S, Lee R, Cho S (2013). Effects of sparse sampling schemes on image quality in low-dose ct. Med. Phys..

[14] Olivo, A. & Speller, R. A coded-aperture technique allowing x-ray phase contrast imaging with conventional sources. *Appl. Phys. Lett.* **91**, 074106 (2007).

[15] Jin KH, McCann MT, Froustey E, Unser M (2017). Deep convolutional neural network for inverse problems in imaging. IEEE Trans. Image Process..

[16] Chen H (2017). Low-dose ct via convolutional neural network. Biomed. Opt. Exp..

[17] Pelt DM, Batenburg KJ, Sethian JA (2018). Improving tomographic reconstruction from limited data using mixed-scale dense convolutional neural networks. J. Imaging.

[18] Adler J, Öktem O (2018). Learned primal-dual reconstruction. IEEE Trans. Med. Imaging.

[19] Lee, H., Lee, J. & Cho, S. View-interpolation of sparsely sampled sinogram using convolutional neural network. In *Medical Imaging 2017: Image Processing*, vol. 10133, 1013328 (International Society for Optics and Photonics, 2017).

[20] Anirudh, R. et al. Lose the views: Limited angle ct reconstruction via implicit sinogram completion. In *Proceedings of the IEEE Conference on Computer Vision and Pattern Recognition*, 6343–6352 (2018).

[21] Yuan, H., Jia, J. & Zhu, Z. Sipid: A deep learning framework for sinogram interpolation and image denoising in low-dose ct reconstruction. In *2018 IEEE 15th International Symposium on Biomedical Imaging (ISBI 2018)*, 1521–1524 (IEEE, 2018).

[22] Lee H, Lee J, Kim H, Cho B, Cho S (2018). Deep-neural-network-based sinogram synthesis for sparse-view ct image reconstruction. IEEE Trans. Radiat. Plasma Med. Sci..

[23] Ghani, M. U. & Karl, W. C. Deep learning-based sinogram completion for low-dose ct. In *2018 IEEE 13th Image, Video, and Multidimensional Signal Processing Workshop (IVMSP)*, 1–5 (IEEE, 2018).

[24] Dong, J., Fu, J. & He, Z. A deep learning reconstruction framework for x-ray computed tomography with incomplete data. *PloS one* **14**, e0224426 (2019). 10.1371/journal.pone.0224426 PMC6824569 31675363

[25] Flenner, S. et al. Pushing the temporal resolution in absorption and zernike phase contrast nanotomography: Enabling fast in situ experiments. J. Synchrotron Radiat. **27** (2020). 10.1107/S1600577520007407 PMC7467338 32876609

[26] Hendriksen AA, Pelt DM, Batenburg KJ (2020). Noise2inverse: Self-supervised deep convolutional denoising for tomography. IEEE Trans. Comput. Imaging.

[27] Hendriksen AA (2021). Deep denoising for multi-dimensional synchrotron x-ray tomography without high-quality reference data. Sci. Rep..

[28] Ronneberger, O., Fischer, P. & Brox, T. U-net: Convolutional networks for biomedical image segmentation. In *International Conference on Medical Image Computing and Computer-Assisted Intervention*, 234–241 (Springer, 2015).

[29] Badrinarayanan V, Kendall A, Cipolla R (2017). Segnet: A deep convolutional encoder-decoder architecture for image segmentation. IEEE Trans. Pattern Anal. Mach. Intell..

[30] Chen L-C, Papandreou G, Kokkinos I, Murphy K, Yuille AL (2017). Deeplab: Semantic image segmentation with deep convolutional nets, atrous convolution, and fully connected crfs. IEEE Trans. Pattern Anal. Mach. Intell..

[31] Pelt DM, Sethian JA (2018). A mixed-scale dense convolutional neural network for image analysis. Proc. Natl. Acad. Sci..

[32] Kampffmeyer, M., Jenssen, R., Salberg, A.-B. et al. Dense dilated convolutions merging network for semantic mapping of remote sensing images. In *2019 Joint Urban Remote Sensing Event (JURSE)*, 1–4 (IEEE, 2019).

[33] Bala, S. A., Kant, S. Dense dilated inception network for medical image segmentation. I*nt. J. Adv. Comput. Sci. Appl.* (2020). 10.14569/IJACSA.2020.0111195.

[34] Takahashi, N. & Mitsufuji, Y. Densely connected multi-dilated convolutional networks for dense prediction tasks. In *Proceedings of the IEEE/CVF Conference on Computer Vision and Pattern Recognition*, 993–1002 (2021).

[35] Pelt, D. et al. Rapid and flexible high-resolution scanning enabled by cycloidal computed tomography and convolutional neural network (cnn) based data recovery (6th International Conference on Image Formation in X-Ray Computed Tomography, 2020).

[36] Zamir, A. *et al.* Recent advances in edge illumination x-ray phase-contrast tomography. *J. Med. Imaging* **4**, 040901 (2017). 10.1117/1.JMI.4.4.040901 PMC5641577 29057286

[37] Bravin A, Coan P, Suortti P (2012). X-ray phase-contrast imaging: from pre-clinical applications towards clinics. Phys. Med. Biol..

[38] Glorot, X. & Bengio, Y. Understanding the difficulty of training deep feedforward neural networks. In *Proceedings of the Thirteenth International Conference on Artificial Intelligence and Statistics*, 249–256 (JMLR Workshop and Conference Proceedings, 2010).

[39] Srivastava N, Hinton G, Krizhevsky A, Sutskever I, Salakhutdinov R (2014). Dropout: a simple way to prevent neural networks from overfitting. J. Mach. Learn. Res..

[40] Ioffe, S. & Szegedy, C. Batch normalization: Accelerating deep network training by reducing internal covariate shift. In *International Conference on Machine Learning*, 448–456 (PMLR, 2015).

[41] Ghodrati V (2019). Mr image reconstruction using deep learning: evaluation of network structure and loss functions. Quant. Imaging Med. Surg..

[42] Pelt, D. M. dmpelt/foam_ct_phantom: 1.1.2 (2020). 10.5281/zenodo.3734782.

[43] Kingma, D. P. & Ba, J. Adam: A method for stochastic optimization. In Bengio, Y. & LeCun, Y. (eds.) *3rd International Conference on Learning Representations, ICLR 2015, San Diego, CA, USA, May 7-9*, 2015, Conference Track Proceedings (2015).

[44] Yang X (2018). Low-dose x-ray tomography through a deep convolutional neural network. Sci. Rep..

[45] Van Aarle W (2016). Fast and flexible x-ray tomography using the astra toolbox. Opt. Express.

[46] Hendriksen, A. et al. Tomosipo: Fast, flexible, and convenient 3d tomography for complex scanning geometries in python. Optics Express (in press, 2021). 10.1364/OE.439909 34809388

[47] Bertels, J. et al. Optimizing the dice score and jaccard index for medical image segmentation: Theory and practice. In *International Conference on Medical Image Computing and Computer-Assisted Intervention*, 92–100 (Springer, 2019).

[48] Wang, Z., Simoncelli, E. P. & Bovik, A. C. Multiscale structural similarity for image quality assessment. In *The Thrity-Seventh Asilomar Conference on Signals, Systems & Computers*, 2003, vol. 2, 1398–1402 (Ieee, 2003).

[49] Paszke, A. et al. Pytorch: An imperative style, high-performance deep learning library. In Wallach, H. et al. (eds.) *Advances in Neural Information Processing Systems* 32, 8024–8035 (Curran Associates, Inc., 2019).

[50] Diemoz, P. *et al.* Single-shot x-ray phase-contrast computed tomography with nonmicrofocal laboratory sources. *Phys. Rev. Appl.* **7**, 044029 (2017).

[51] Paganin D, Mayo SC, Gureyev TE, Miller PR, Wilkins SW (2002). Simultaneous phase and amplitude extraction from a single defocused image of a homogeneous object. J. Microsc..

[52] Roche i Morgó, O., Vittoria, F. A., Endrizzi, M., Olivo, A. & Hagen, C. K. Technical note: Practical implementation strategies of cycloidal computed tomography. Med. Phys. (in press). 10.1002/mp.14821 PMC11497279 34169514

[53] Guizar-Sicairos M, Thurman ST, Fienup JR (2008). Efficient subpixel image registration algorithms. Opt. Lett..

